# Supplementary material for: Predictors of Length of Stay, Mortality and Rehospitalization in COPD Patients: A Retrospective Cohort Study
Source: J Clin Med. 2023 Aug 16;12(16):5322. doi: 10.3390/jcm12165322 (PMC10455093; doi:10.3390/jcm12165322)
Supplement: Supplementary file 1 [file jcm-12-05322-s001.zip › jcm-2513760-supplementary.pdf]

Supplementary Materials:

Figure S1. Graph of one year mortality by age groups.

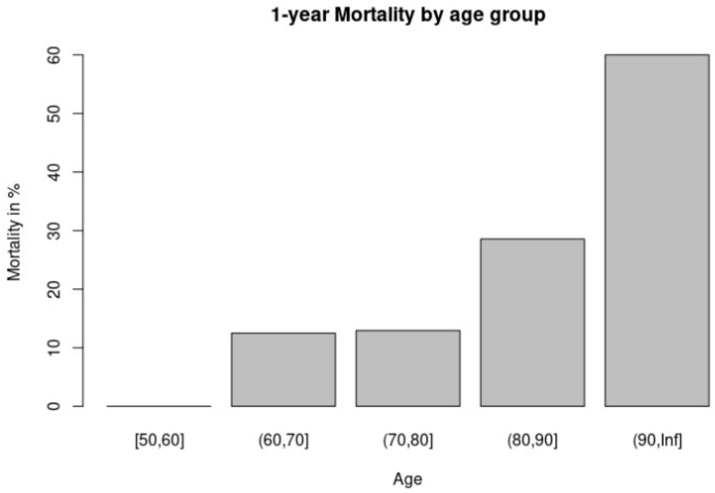

Table S1. Sensitivity analysis. Results of multivariable zero-truncated negative binomial regression model for length of hospital stay (LOHS) estimation in AECOPD survivors (*n* =164) controlling for pneumonia.

	LOHS Prediction	IRR	(95%CI)		*p*-Value
(Intercept)	8.333	5.33	2.683	10.59	0.000
Sex	7.808	0.936	0.797	1.099	0.421
Age	8.384	1.006	0.997	1.015	0.169
Severe COPD (GOLD III or IV)	7.931	0.951	0.809	1.119	0.546
Heart failure	7.758	0.93	0.698	1.239	0.62
Ischemic heart disease	7.369	0.883	0.742	1.049	0.157
Arrhythmias	7.726	0.926	0.756	1.134	0.459
Peripheral artery disease (PAD)	9.413	1.131	0.918	1.392	0.247
Diabetes	7.691	0.922	0.757	1.123	0.42
Asthma-COPD overlap	8.638	1.037	0.832	1.292	0.747
Active cancer	6.613	0.79	0.608	1.027	0.078
Ambulance transportation	8.933	1.073	0.913	1.261	0.395
**Indication for oxygen supplementation**	**10.962**	**1.318**	**1.126**	**1.542**	**0.001**
CRP	8.339	1.001	1	1.002	0.231
Leucocytosis	7.47	0.895	0.767	1.045	0.16
Eosinophils	5.715	0.679	0.412	1.121	0.13
Pneumonia	6.898	0.825	0.666	1.022	0.079

Table S2. Sensitivity analysis. Results of multivariable logistic regression model for one year mortality in patients with COPD controlling for pneumonia.

	OR	(95%CI)		*p*-Value
Sex	0.465	0.132	1.532	0.216
**Age**	**1.104**	**1.029**	**1.193**	**0.008**
**Severe COPD (GOLD III or IV)**	**4.527**	**1.348**	**19.225**	**0.024**
Heart failure	0.825	0.091	4.67	0.842
Ischemic heart disease	0.62	0.158	2.104	0.461
Arrhythmias	7.88	2.426	28.768	0.001
Peripheral artery disease (PAD)	0.76	0.151	3.108	0.717
Diabetes	1.981	0.548	6.947	0.285
Asthma-COPD overlap	0.661	0.079	3.522	0.658
**Active cancer**	**8.084**	**2.096**	**33.66**	**0.003**
**Rehabilitation**	**0.07**	**0.002**	**0.604**	**0.048**
Length of hospital stay	0.934	0.812	1.064	0.313
Pneumonia	1.205	0.296	4.372	0.782
